# Mississippi Valley Med. Asso.

**Published:** 1889-11

**Authors:** 


MISSISSIPPI VALEY MEDICAL ASSOCIATION.—We are indebted to the kindness of Dr. E. S. McKee of Cincinnati for the following.

The 15th annual session of the Mississippi Valley Medical Asssociation was held at Evansville, Indiana, Sept. 10, 11 and 12, 1889. The attendance was excellent and an immense programme, about 100 papers were presented.

The President, Dr. Geo. F. Cook, of Indianapolis, made a few opening remarks. Dr. Owen, of Evansville, the very efficient chairman of the committee of arrangements, welcomed the visiting physicians on behalf of the City of Evansville and the practitioners of that place.

The meeting was a very successful one from the number of papers presented, and the depth of research and learning displayed by the authors. The discussions were interesting. While the day was thus profitably spent, the social feature of the meeting was an important part of the occasion. On the evening of the first day a banquet was given the Society in a beautiful grove. About 500 doctors and citizens with ladies, enjoyed the feast of reason and flow of soul, which under the leadership of Dr. I. N. Love, of St. Louis, as toast master, made Garvin Park re-echo again and again. A complimentary ball and concert were given the second evening which was a most enjoyable affair. An excursion to Newberg occupied the third evening; besides these were a number of other private entertainments, so Evansville covered herself with glory in the efforts for the pleasure of the meeting within her gates.

A number of prominent gentlemen from without the Mississippi Valley were present. Among them were Drs. Geo. H. Rohe, of Baltimore, and W. C. Wile, of Danbury Conn. The latter decorated some of the members with the badge of the Order of Connecticut, wooden nutmegs attached to the coat button by a green ribbon. Dr. Rohe's mention of the fact of the Star Spangled Banner being written in his own city, produced a patriotic scene.

The Committee on Nominations, reported by their Chairman, Dr. I. N. Love: President, Dr. Joseph M. Matthews, Louisville; First Vice President, Dr. C. R. Early, Pennsylvania; Second Vice President, Dr. T. B. Harvey, Indianapolis, Permanent Secretary; Dr. E. S. McKee, Cincinnati, Treasurer. Louisville was chosen as the next place of meeting, Sept. 9, 10 and 11, 1890. Dr. J. N. Bloom, of Louisville was elected chairman of Committee of Arrangements.

